# Morphological differences between residual childhood hip dysplasia with previous osteotomy and adolescent-onset hip dysplasia

**DOI:** 10.1186/s13018-025-05655-w

**Published:** 2025-03-13

**Authors:** Han-Jin Liu, I-Hsin Chen, Ting-Ming Wang, Chia-Che Lee, Sheng-Chieh Lin, Ken N. Kuo, Kuan-Wen Wu

**Affiliations:** 1 Department of Orthopaedic Surgery, National Taiwan University Hospital, No. 7, Chung Shan S. Road, Zhongzheng District, Taipei City, 10002 Taiwan; 2 Graduate Institute of Biomedical Electronics and Bioinformatics, National Taiwan University, Taipei City, Taiwan; 3 Department of Orthopaedic Surgery, School of Medicine, National Taiwan University, Taipei City, Taiwan; 4 Department of Orthopaedic Surgery, Chung Shan Medical University Hospital, Taipei City, Taiwan; 5 Cochrane Taiwan, Taipei Medical University, Taipei City, Taiwan

**Keywords:** Hip dysplasia, Morphological analysis, Periacetabular osteotomy

## Abstract

**Background:**

Hip dysplasia (HD) at skeletal maturity can result from residual developmental dysplasia of the hip (DDH) treated in childhood or from primary adolescent-onset HD (AOHD). This study aims to compare the pathomorphology of these two HD subtypes with that of a normal control group.

**Methods:**

This retrospective study reviewed patients who underwent periacetabular osteotomy for symptomatic HD between 2013 and 2020. The study included 27 residual HD patients (32 hips) following a previous pelvic osteotomy and 39 AOHD patients (68 hips), compared to 29 age- and sex-matched healthy individuals. Acetabular morphology was assessed using plain radiographs, measuring the lateral and anterior center-edge angle (LCEA/ACEA), Sharp angle, Tönnis angle (TA), acetabular depth ratio (ADR), acetabular head index (AHI), and head lateralization index (HLI). On 2D axial and frontal CT scans, we measured acetabular version (AV), anterior and posterior acetabular sector angle (AASA/PASA), femoral neck shaft angle (NSA) and femoral anteversion (FAV).

**Results:**

Both HD groups presented frontal and sagittal acetabular dysplasia with lower LCEA (*p* < 0.001), lower ACEA (*p* < 0.001), and lateral subluxation, indicated by lower AHI (*p* < 0.001) and higher HLI (*p* < 0.001). Compared to AOHD, residual HD demonstrated greater lateralization, with a higher HLI (*p* = 0.028). In the axial plane, both HD groups had similar deficient anterior coverage, with lower AASA (*p* < 0.001). However, residual HD exhibited poorer posterior coverage, with a lower PASA (*p* < 0.001) and a lower AV (*p* = 0.006). NSA did not differ between groups, but residual HD had excessive FAV compared to the other groups (*p* < 0.001).

**Conclusions:**

Although both residual HD and AOHD demonstrated anterior and lateral acetabular deficiencies, residual HD was further characterized by reduced acetabular version, more femoral head lateralization, poorer posterior acetabular support, and excessive FAV.

## Background

Hip dysplasia (HD) is a general term describing inadequate acetabulum coverage of the unstable femoral head and associated with several genetic deficiencies [1–3]. There are two types of HD depending on the time of occurrence [4]. Residual HDs are a late presenting form of HD following previous treated developmental dysplasia of the hip (DDH) [4]. The other type, adolescent-onset HDs (AOHD) are those with stable hips at birth, then, later presented symptomatic shallow acetabulum in adolescence [5]. Although both types elicit a similar mechanical disadvantage of the hip joint, it has been postulated that AOHD may be a different disease from childhood DDH [4, 5]. For both types of HD, the treatment at or near skeletal maturity is to restore adequate coverage to minimize the risk of developing early osteoarthritis.

The Bernese periacetabular osteotomy (PAO) has become the treatment of choice for correcting symptomatic HD [6–8], as several other osteotomies for DDH and Perthes disease [9], by allowing the reorientation of dysplastic acetabulum in multiple directions to achieve an optimal correction, but can be challenging. The PAO for treating residual HD can be more complex than AOHD due to previous osteotomy-related deformities [10–12]. In order to maximize the success of PAO, an understanding of pathoanatomical properties of dysplastic hips with multi-planar imaging assessments is mandatory, and so is true for rotational acetabular osteotomy [13]. With recent advances in image processing, various patterns of HD have been identified [14–16]. Several studies were reported on hip morphologies, however, these studies included patients with a mixture of juvenile and adult cases or excluded previous pelvic osteotomies cases [17–19].

The purpose of our study is to compare the different morphological features of the hips between the residual HD group, the AOHD group, and the normal group in terms of [1] severity of frontal and sagittal acetabular dysplasia and lateral subluxation [2] axial acetabular osseous support and acetabular version (AV), and [3] degree of coxa valga and femoral anteversion (FAV) at the time of PAO.

## Methods

### Study design and patients

This retrospective study received institutional review board approval (202205007RINC) with waiver of informed consent. All procedures followed institutional and national research committee ethical standards, as well as the 1964 Helsinki declaration and its later amendments.

At our institution, we identified 110 consecutive patients (157 hips) who underwent PAO for HD between 2013 and 2020 due to persistent symptoms despite conservative treatments, with radiographic evidence of a lateral center-edge angle (LCEA) < 20°. Patients with HD secondary to syndromic etiologies (2 hips), neuromuscular disease (12 hips), skeletal disorders (14 hips), and concomitant femoral procedures (5 hips) for proximal femoral deformities were excluded. In the AOHD group, we also excluded 24 hips that were outside the age range of the residual HD group. The final study cohort consisted of 100 hips in 66 patients. For comparison, we selected 29 age- and sex-matched normal adults (30 hips) who had undergone imaging for reasons unrelated to hip dysplasia (Table 1).

**Table 1:** Demographic data in three study groups

	Residual HD (*n* = 32)	AOHD (*n* = 68)	Control (*n* = 30)	*p* value
Sex (male/female)	4/23	6/33	5/24	0.966
Laterality (R/L/B)	8/14/10	3/7/58	21/7/2	0.000
Age (years)	19.5 ± 7.6	24.6 ± 9.3	23.0 ± 10.4	0.088

### Imaging protocol and analysis

All subjects had preoperative standing anteroposterior (AP) pelvis radiographs, false-profile views, and a thin-slice computed tomography (CT) scan of the pelvis. Imaging was performed with the legs fully extended and the knees positioned straightforward.

### Radiographic parameters measurement

**Fig. 1:**
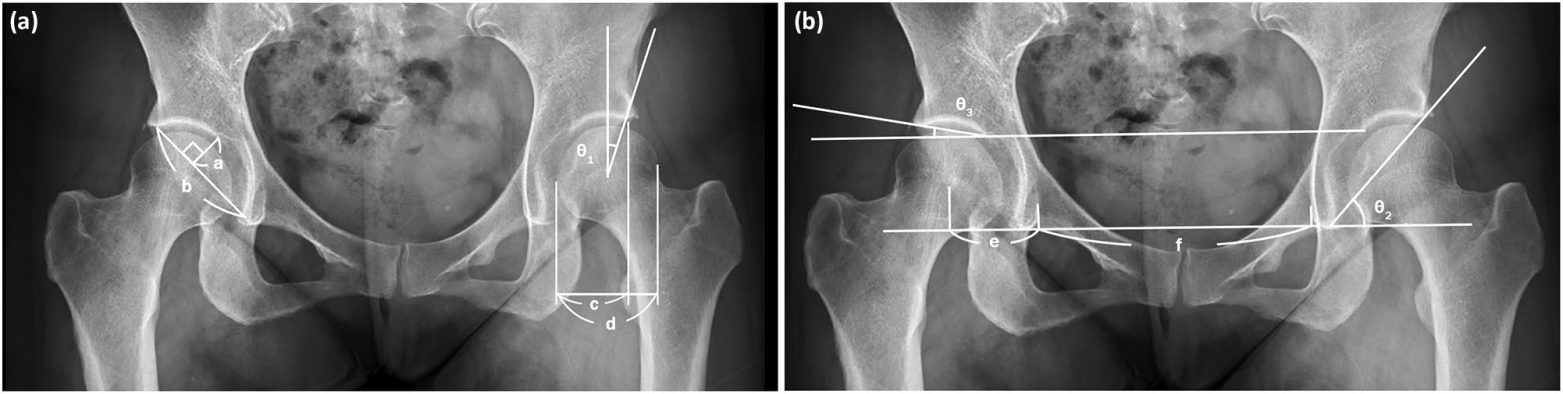
(**A**) Parameters used to measure acetabular dysplasia and lateral subluxation. θ1 is the lateral CE angle (LCEA). a/b is the acetabular depth ratio (ADR). c/d is the acetabulum head index (AHI). These parameters were measured on the AP radiograph. CE angle was measured as the angle between the line joining lateral aspect of the weight-bearing zone and the femoral head center and the line running vertically along the longitudinal axis of the pelvis. ADR was calculated by dividing the depth of the acetabulum by the length between the inferior teardrop point and the aspect of the weight-bearing zone, then multiplying by 100. AHI was calculated by dividing the length from the medial margin of the femoral head to the lateral rim of the acetabulum by the femoral head width, then multiplying by 100. (**B**) Parameters used to measure acetabular dysplasia and lateral subluxation. θ2 is the Sharp angle. θ3 is the acetabular roof obliquity angle of Tönnis (TA). e/(f*1/2) is the head lateralization index (HLI) described by Ninomiya S. These parameters were measured on the AP radiograph. Sharp angle was measured as the angle between the line joining the lateral aspect of the weight-bearing zone and the inferior point of teardrop and the line joining bilateral inferior points of teardrop. Acetabular roof obliquity angle of Tönnis was measured as the angle between the line joining the lateral aspect of the weight-bearing zone and the medial aspect of the weight-bearing zone and the line joining bilateral medial aspects of the weight bearing zone. HLI was calculated by dividing the length from the medial margin of the teardrop and the femoral head center by half of the length between bilateral medial margins of the teardrop

**Fig. 2:**
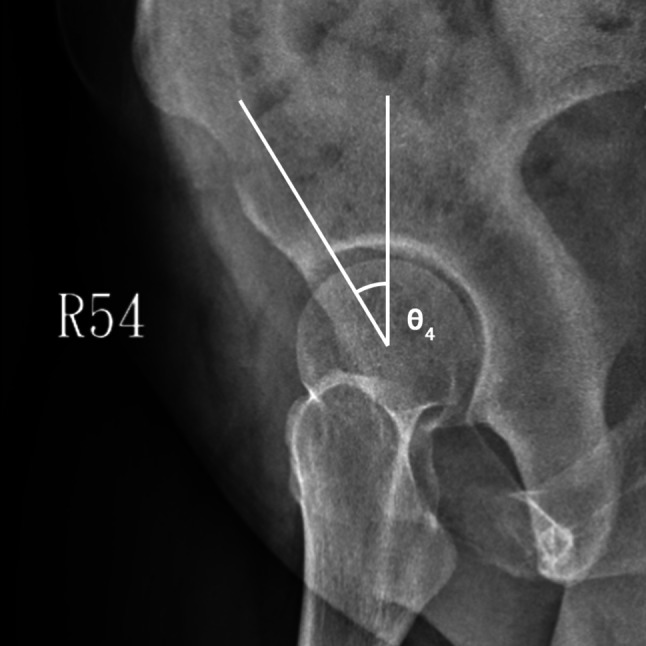
Parameters used to measure acetabular dysplasia and lateral subluxation. θ4 is the anterior CE angle (ACEA) described by Lequesne M. This parameter was measured on the false-profile view. ACEA was measured as the angle between the line joining asterior aspect of the weight-bearing zone and the femoral head center and the line running vertically along the longitudinal axis of the pelvis

**Fig. 3:**
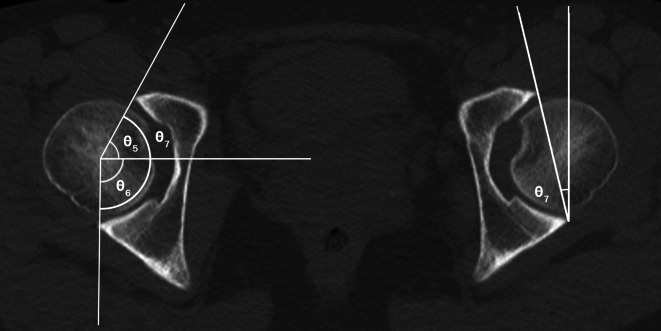
Parameters used to measure acetabular version and axial osseous support. θ5 and θ6 are the anterior acetabular sector angle (AASA) and posterior acetabular sector angle (PASA), respectively. θ7 are the horizontal acetabular sector angle (HASA). θ8 is the acetabular version (AV) described by Anda S. These parameters were measured on were measured on axial images of CT scan at the level of the femoral head center. AASA was measured as the angle between the inter-capital centerline and the line joining the anterior margin of the acetabulum and the femoral head center. PASA was measured as the angle between the inter-capital centerline and the line joining the posterior margin of the acetabulum and femoral head center. HASA was calculated as the summation of AASA (θ5) and PASA (θ6). AV was measured as the angle between the line connecting the anterior and posterior margins of the acetabulum and a line perpendicular to the inter-capital centerline

**Fig. 4:**
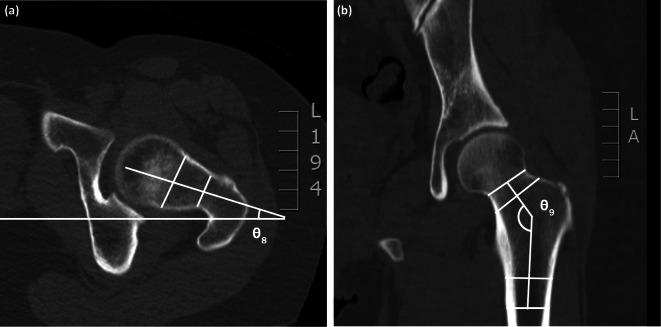
(**A**) Parameters used to measure coxa valga and the femoral anteversion. θ9 is femoral neck anteversion (FAV) described by Perreira AC. This parameter was measured on the axial images of CT scan. FAV was measured as the angle between a line bisecting the long axis of the femoral neck (from the center of the femoral head to center of the femoral neck) and a line parallel to the distal femoral posterior condylar axis. (**B**) Parameters used to measure coxa valga and the femoral anteversion. θ10 is the neck shaft angle (NSA). This parameter was measured on the coronal images of CT scan. NSA was measured as the angle between a line bisecting the long axis of the femoral neck and a line bisecting the long axis of the femoral shaft

On radiographs, we assessed acetabular dysplasia by measuring the LCEA, Sharp angle, acetabular roof obliquity Tönnis angle (TA), acetabular depth ratio (ADR) on AP view (Fig. 1A and B), as well as the anterior center-edge angle (ACEA) on the false-profile radiographs (Fig. 2). We also measured the acetabular head index (AHI) [20] and head lateralization index (HLI) [21] to evaluate femoral head coverage and femoral head lateralization (Fig. 1A and B). On 2D axial and frontal CT scans, we assessed axial acetabular osseous support, acetabular version (AV), femoral neck shaft angle (NSA), and neck anteversion (FAV). To evaluate anterior and posterior acetabular bony support of the femoral head, we measured the anterior acetabular sector angle (AASA) and the posterior acetabular sector angle (PASA) on axial images at the level of the femoral heads center (Fig. 3) [22]. Additionally, AV angle was measured on axial CT scans (Fig. 3) [22], while FAV was determined as the angle between a line bisecting the long axis of the femoral neck and a horizontal line on axial CT scans (Fig. 4A) [23]. Femoral NSA was measured on a frontal-plane CT scan at the level of the femoral head center (Fig. 4B) [24]. Regarding complications, in the residual HD group, radiographic signs of osteonecrosis (ON) of the proximal femoral epiphysis were recorded using the Kalamchi classification [25].

### Statistical analysis

For analyzing intra-rater and inter-rater reliability, intraclass correlation coefficient (ICC) analysis was performed. Intra-rater reliability was evaluated by repeating the measurement one-week apart by the same author (HJL). For inter-rater reliability, another coauthor (CCL) independently measured all the parameters using the same software and methods. The ICC values demonstrated good-to-excellent intra-rater and inter-rater reliability (Table 2).

**Table 2:** Intraclass correlation coefficient values for each radiological measurement

category	parameters	intra-rater ICCs (95% CI)	inter-rater ICCs (95% CI)
Acetabular dysplasia and lateral subluxation	LCEA	0.993 (0.984, 0.997)	0.987 (0.984, 0.997)
	ACEA	0.985 (0.968, 0.993)	0.989 (0.977, 0.995)
	Sharp angle	0.985 (0.968, 0.993)	0.979 (0.954, 0.990)
	Tönnis angle	0.995 (0.990, 0.998)	0.984 (0.963, 0.993)
	ADR	0.982 (0.960, 0.991)	0.965 (0.925, 0.984)
	AHI	0.995 (0.988, 0.998)	0.847 (0.631, 0.933)
	HLI	0.979 (0.954, 0.990)	0.945 (0.880, 0.975)
Acetabular version and axial osseous support	AASA	0.993 (0.985, 0.997)	0.989 (0.977, 0.995)
	PASA	0.991 (0.981, 0.996)	0.983 (0.964, 0.992)
	Acetabular version	0.999 (0.997, 0.999)	0.997 (0.994, 0.999)
coxa valga and femoral anteversion	NSA	0.981 (0.959, 0.991)	0.934 (0.858, 0.969)
	FAV	0.998 (0.996, 0.999)	0.990 (0.979, 0.996)

Data are reported as interobserver and intraobserver correlation coefficient with 95% confidence interval; LCEA = lateral center-edge angle, ACEA = anterior center-edge angle, AI = acetabular index, ADR = acetabular depth ratio, AHI = acetabular head index, HLI = head lateralization index, AASA = anterior acetabular sector angle, PASA = posterior acetabular sector angle, FNSA = femoral neck shaft angle, FNA = femoral neck anteversion

Data are reported as interobserver and intraobserver correlation coefficient with 95% CI

Continuous variables were presented as the mean ± SD. All continuous variables were first examined for normality using the Shapiro-Wilk test. If the data were normally distributed, differences in demographic and radiographic indices among the three study groups were determined using analysis of variance (ANOVA) with a post hoc Tukey test for continuous variables. If data were not non-normally distributed, the Kruskal–Wallis test was used to analyze mean differences. Pairwise differences between groups were determined using the Mann-Whitney U test. Differences in the sex and laterality between groups were analyzed using χ^2^ test. All statistical analyses were performed using SPSS software (version 26.0, SPSS Inc, Chicago, IL, USA). A p-value < 0.05 was considered statistically significant. The 95% confidence interval (CI) were reported when applicable.

## Results

### Study population

Among the study groups, there were 32 hips in 27 patients (mean age: 19.5 years, range 12–42) classified as residual HD who had undergone open reduction and pelvic osteotomy for childhood DDH. The other 68 hips in 39 patients (mean age: 24.6 years) comprised the AOHD group. There was no significant difference in sex distribution (*p* = 0.966) and mean age (*p* = 0.088) between the residual HD group, the AOHD group and the comparison group (Table 1).

### Frontal and sagittal acetabular dysplasia

Both the residual HD group and the AOHD group presented apparent acetabular dysplasia compared to normal by plain AP and false-profile radiographs (Table 3). Radiographic analysis revealed that both HD groups had significantly lower mean LCEA, ACEA, and ADR, along with higher mean Sharp angle and TA compared to the normal group (*p* < 0.001). Additionally, the residual HD group had a lower mean Sharp angle and ADR than the AOHD group with statistical significance (*p* = 0.001 and *p* = 0.009). However, there were no significant differences in LCEA, ACEA, or TA between the two HD groups (Table 3).

**Table 3:** Comparisons of frontal and saggital acetabular dysplasia between three groups

	Residual HD (*R*) (95% CI)	AOHD (A) (95% CI)	Control (C) (95% CI)	*p* value	Post Hoc (Turkey)					
					*R*-A (95% CI)	*p* value	*R*-C (95% CI)	*p* value	A-C (95% CI)	*p* value
LCEA (°)	5.3 ± 14.1 (0.3, 10.4)	7.3 ± 9.8 (4.9, 10.7)	26.4 ± 4.8 (24.6, 28.2)	0.000	-2.0 (-7.2, 3.2)	0.637	-21.1 (-27.3, -14.9)	0.000	-19.1 (-24.4, -13.8)	0.000
ACEA (°)	10.5 ± 13.5 (5.5, 15.6)	10.4 ± 11.8 (7.6, 13.3)	29.9 ± 6.4 (27.2, 32.5)	0.000	0.1 (-5.8, 6.0)	0.999	-19.3 (-26.7, -12.0)	0.000	-19.5 (-25.8, -13.1)	0.000
Sharp angle (°)	47.2 ± 6.0 (45.0, 49.3)	50.8 ± 4.5 (49.7, 51.9)	44.2 ± 3.3 (42.9, 45.4)	0.000	-3.6 (-6.0, -1.2)	0.001	3.0 (0.1, 5.8)	0.037	6.6 (4.2, 9.0)	0.000
Tönnis angle (°)	19.8 ± 9.9 (16.3, 23.4)	22.4 ± 9.4 (20.1, 24.7)	7.3 ± 4.3 (5.7, 8.9)	0.000	-2.5 (-6.9, 1.8)	0.358	12.6 (7.4, 17.8)	0.000	15.1 (10.6, 19.6)	0.000
ADR (%)	21.9 ± 5.0 (20.1, 23.7)	24.9 ± 5.0 (23.7, 26.1)	31.7 ± 3.4 (30.5, 33.0)	0.000	-3.0 (-5.4, -0.6)	0.009	-9.8 (-12.6, -7.0)	0.000	-6.8 (-9.2, -4.4)	0.000

Data are presented as mean ± standard deviation (95% confidence interval)

LCEA = lateral center-edge angle, ACEA = anterior center-edge angle, ADR = acetabular depth ratio

Data are presented as the mean ± SD (95% CI)

### Femoral head coverage and lateral subluxation

Both HD groups showed insufficient femoral head coverage and lateral subluxation as indicated by a significantly lower mean AHI (*p* < 0.001) and a significantly higher mean HLI (*p* < 0.001 and *p* = 0.002) compared to the normal group. Between the two HD groups, the AHI values were similar; however, the residual HD group had a significantly greater mean HLI than the AOHD group, indicating that the center of the femoral head was shifted further laterally from the midline (95% CI of the difference, 0.6. to 12.6; *p* = 0.028) (Table 4).

**Table 4:** Comparisons of frontal femoral head coverage and lateralization between three groups

	Residual HD (*R*) (95% CI)	AOHD (A) (95% CI)	Control (C) (95% CI)	*p* value	Post Hoc (Turkey)					
					*R*-A (95% CI)	*p* value	*R*-C (95% CI)	*p* value	A-C (95% CI)	*p* value
AHI (%)	60.5 ± 15.5 (54.9, 66.1)	60.4 ± 12.6 (57.3, 63.4)	77.8 ± 6.4 (75.5, 80.2)	0.000	0.1 (-6.1, 6.4)	0.999	-17.3 (-24.8, -9.9)	0.000	-17.5 (-23.9, -11.0)	0.000
HLI (%)	79.5 ± 15.6 (73.9, 85.1)	72.9 ± 10.2 (70.5, 75.4)	63.9 ± 10.4 (60.1, 67.8)	0.000	6.6 (0.6, 12.6)	0.028	15.6 (8.5, 22.7)	0.000	9.0 (2.9, 15.2)	0.002

Data are presented as mean ± standard deviation (95% confidence interval)

AHI = acetabular head index, HLI = head lateralization index

AHI = acetabular head index

HLI = head lateralization index

Data are presented as the mean ± SD (95% CI)

### Axial acetabular osseous support, acetabular version and proximal femoral alignment

In terms of axial acetabular osseous support, both HD groups exhibited deficient anterior osseous support compared to the normal group with significantly lower mean AASA (*p* < 0.001). However, there was no significant difference in AASA between the two HD groups (Table 5). In contrast, the residual HD group demonstrate worse posterior osseous support, with a significantly lower mean PASA compared to both the AOHD and normal group (*p* < 0.001). Although the AOHD group also had a significantly lower PASA compared to the normal group (*p* = 0.044) (Table 5), the deficiency was more pronounced in the residual HD group. Regarding acetabular version (AV), there was no significant difference in mean AV between each HD group and the normal group. However, when comparing the two HD groups, the residual HD group exhibited a significantly lower mean AV than the AOHD group (*p* = 0.006). There was no significant difference in mean femoral NSA among the three groups. However, regarding femoral anteversion (FAV), the residual HD group had a significantly greater mean FAV compared to both the AOHD and normal group (*p* < 0.001) (Table 5). Among childhood DDH cases, 22 of 32 hips (68.8%) exhibited femoral head ON, including 3 Kalamchi type I, 17 type II, 1 type III and 1 type IV.

**Table 5:** Comparisons of axial acetabular version, osseous support, and proximal femoral alignment between three groups

	Residual HD (*R*) (95% CI)	AOHD (A) (95% CI)	Control (C) (95% CI)	*p* value	Post Hoc (Turkey)					
					*R*-A (95% CI)	*p* value	*R*-C (95% CI)	*p* value	A-C (95% CI)	*p* value
AASA (°)	43.7 ± 13.8 (38.6, 48.7)	44.7 ± 8.2 (42.7, 46.6)	57.0 ± 7.7 (54.0, 59.9)	0.000	-1.0 (-6.0, 4.0)	0.886	-13.3 (-19.2, -7.3)	0.000	-12.3 (-17.4, -7.2)	0.000
PASA (°)	77.1 ± 10.8 (73.1, 81.1)	88.6 ± 8.0 (86.7, 90.6)	93.6 ± 9.9 (89.8, 97.3)	0.000	-11.5 (-16.2, -6.8)	0.000	-16.4 (-22.1, -10.8)	0.000	-4.9 (-9.8, -0.1)	0.044
Acetabular Version (°)	17.3 ± 9.9 (13.7, 20.9)	22.2 ± 5.9 (20.8, 23.6)	18.9 ± 6.3 (16.5, 21.3)	0.004	-4.9 (-8.6, -1.2)	0.006	-1.6 (-6.0, 2.8)	0.664	3.3 (-0.5, 7.0)	0.099
NSA (°)	139.4 ± 10.2 (135.7, 143.0)	138.1 ± 6.9 (136.4, 139.8)	134.8 ± 5.3 (132.8, 136.8)	0.047	1.3 (-2.5, 5.1)	0.710	4.6 (0.0, 9.1)	0.470	3.3 (-0.6, 7.2)	0.115
FAV (°)	29.7 ± 19.2 (22.7, 36.8)	12.0 ± 14.0 (8.7, 15.4)	9.7 ± 15.0 (4.03, 15.4)	0.000	17.7 (9.7, 25.7)	0.000	20.0 (10.5, 29.6)	0.000	2.3 (-5.9, 10.5)	0.783

Data are presented as mean ± standard deviation (95% confidence interval)

AASA = anterior acetabular sector angle, PASA = posterior acetabular sector angle, NSA = femoral neck shaft angle, FAV = femoral neck anteversion

Data are presented as the mean ± SD (95% CI)

## Discussion

### Main findings

To the best of our knowledge, the geometry of the residual HD following previous pelvic osteotomy for childhood DDH has not been thoroughly investigated. To enhance Bernese PAO surgical planning, we analyzed 3D morphological features of the hip in two common adult HD types, using matched normal adults as a control group. Our findings reveal distinct morphological patterns in residual HD compared to AOHD, including significantly lower acetabular volume, greater femoral head lateralization, poorer posterior acetabular support, and excessive femoral anteversion.

### Comparison with literature

#### Frontal and sagittal acetabular dysplasia measurements

**Fig. 5:**
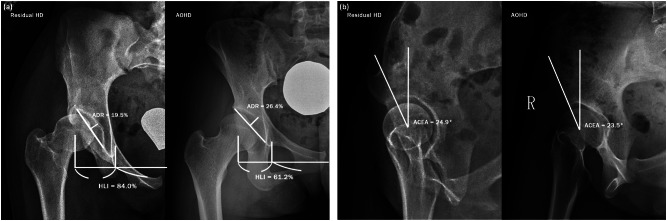
(**A**) Comparison of the AP radiographs of the sample images from the residual HD group (left) and the AOHD group (right). (**B**) Comparison of the false-profile view sample images from the residual HD group (left) and the AOHD group (right)

Comparison of the radiographic measurements supports the notion that both HD groups exhibited apparent frontal and sagittal acetabular dysplasia at the time of the PAO procedure. The severity of both HD groups met the criteria of moderate to severe dysplasia (LCEA < 15°, ACEA < 15°, Sharp angle > 45°, TA > 10°, and ADR < 25%) as described in previous studies [15, 26], with similar severity (Fig. 5A and B) except for a significantly lower acetabular volume in the residual HD group compared to the AOHD group, as measured by ADR. Despite similar abnormalities in anatomical structures, these two groups affect the growing hips at different periods with different pathomechanisms. For AOHD, it has been theorized that delayed ossification of the triradiate cartilage and insufficient development of the lateral secondary ossification centers of the acetabulum during adolescence [4] contribute to the condition. This explains why lateral acetabular roof deficiency is the major feature in AOHD along with varying degrees of anterosuperior or posterior deficiency [14–16].

Residual HD resulted from childhood DDH can also present in the young adulthood. Modaressi et al. reported a 2.7% (4/150) incidence of residual dysplasia at puberty after successful treatment of infant DDH [27]. All of our residual HD patients underwent open reduction and Pemberton osteotomy for DDH at the walking age. The relatively lower TA and significantly lower Sharp angle in residual HD might be the result of prior Pemberton osteotomy.

It remains unclear why some residual HD cases, despite initially achieving good acetabular coverage following the procedure, later develop dysplasia at puberty—even in those without femoral head ON. During early development, the normal physiological growth and deepening of the hip joint rely on the reciprocal interaction between the femoral head and the acetabulum. The appearance of the femoral head ossification center in DDH is often delayed, relatively smaller and eccentrically located [28]. Any disruption to the hip’s normal congruency after surgery or late reduction may cause acetabular floor hyperplasia resulting from the asymmetrical interaction between the ball-and-socket joint, leading to a secondary shallow acetabulum at skeletal maturity [29].

We hypothesize that different underlying pathomechanisms contribute to the significantly lower ADR in residual HD compared to AOHD. While routine pelvic AP plain film might be useful for evaluating the frontal position of hip joint, they have limitations in assessing the adequacy of 3D concentricity between the acetabulum and femoral head. A 3D imaging modality is preferred to confirm the accurate restoration of the hip joint after treatments and to identify potential obstacles to concentric reduction at an early stage if unsuccessful [30].

#### Femoral head coverage and lateral subluxation

We determined the severity of hip subluxation by measuring AHI and HLI (Fig. 1A and B). The general criteria for HD is defined as AHI < 75% [26]. Both HD groups demonstrated a similar severity of a significantly deficient femoral head coverage, with an average of AHI 60%, compared to 77.8% in the control group (Fig. 5A and B). The decreased femoral head coverage may result not only from the underdevelopment of acetabular roof but also the lateralization of the femoral head. Since the femoral head in dysplastic hips tends to shift laterally, both HD groups exhibited a significantly higher HLI compared to the control group (79.5% & 72.9% versus 63.9%). However, between the two HD groups, residual HD demonstrated a significantly higher HLI than AOHD, indicating a more laterally displaced femoral head.

In post-treatment DDH cases, increased acetabular floor thickness and an enlarged femoral head are often observed at an early stage and persisted despite satisfactory DDH reduction [31]. Among the 22 hips with ON in our study, 17 (77.2%) were classified as Kalamchi type II ON. Type II ON is the most common type of this growth disturbance [25, 32]. The subsequent valgus deformity from premature lateral physeal closure may lead to lateral tilting of the femoral head. This underscores the importance of medializing the joint center in residual HD cases when performing the PAO.

#### Axial acetabular osseous support, acetabular version and proximal femoral alignment

**Fig. 6:**
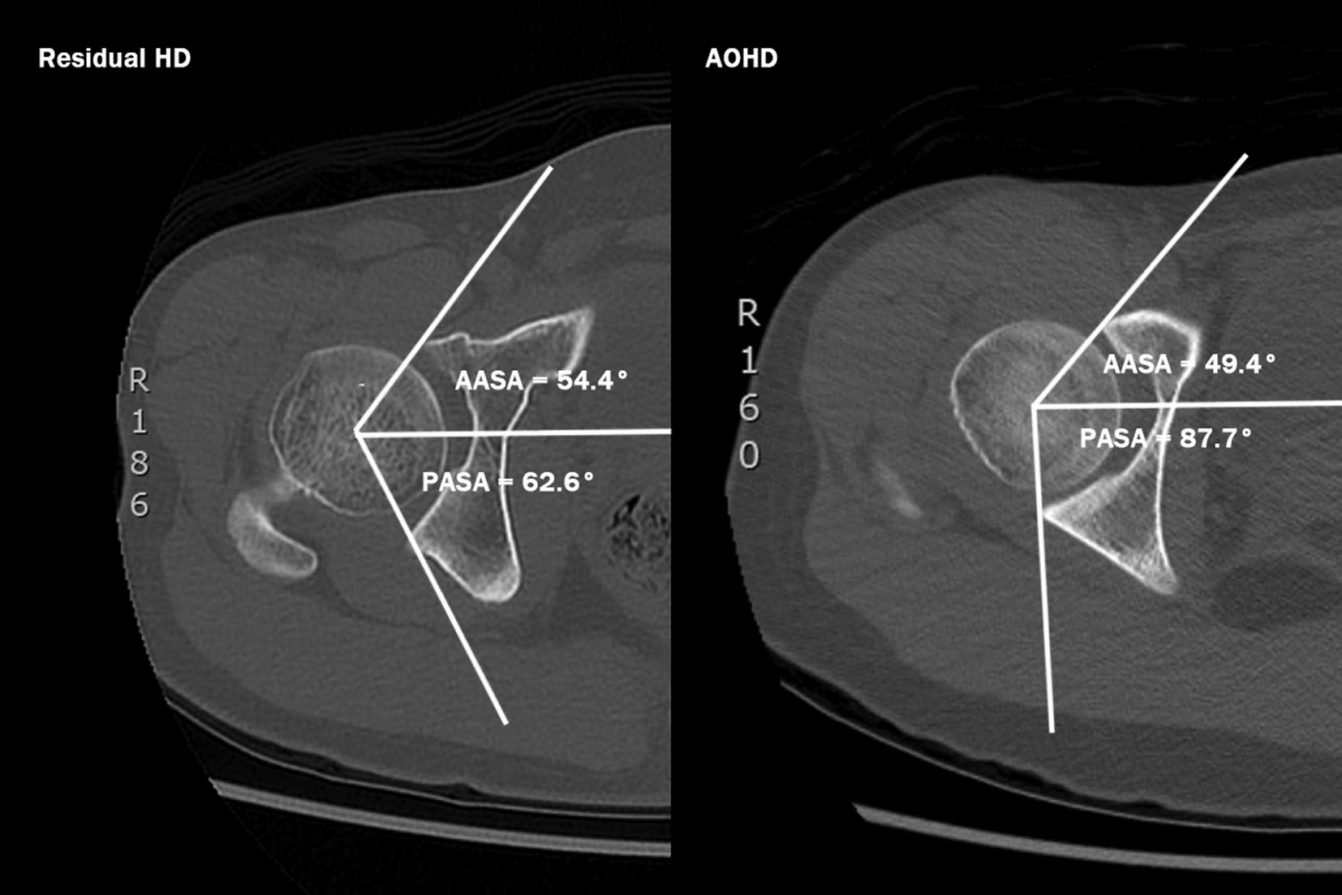
Comparison of the axial images of the sample images from the residual HD group (left) and the AOHD group (right) at the level of femoral head

While plain radiographs demonstrate superolateral deficiency in most HD patients, axial 2D-CT scans allow for a more precise quantification of acetabular orientation abnormalities and identification of acetabular defects in the anterior and posterior walls [17, 22, 26]. Anda et al. first described acetabular sector angles on axial CT scans to distinguished axial acetabular support from acetabular coverage [22]. Mean values for the AASA and PASA in a general Asian population-based survey were reported as 59.3° and 98.6°, respectively [26]. These values are typically reduced in patients with HD, especially in those with anterior coverage deficiency [17]. In our study, the mean values of AASA were significantly lower in both HD groups compared to the control group, though no significant difference was found between the two HD groups. However, for PASA, both HD groups exhibited decreased mean values, with the residual HD group significantly lower than the AOHD group (Fig. 6). The significantly decreased AASA and PASA indicated that global acetabular deficiency persisted in residual HD in spite of previous corrective surgery. This information is crucial for PAO planning, as surgical correction should be tailored to the specific acetabular deficiencies to ensure comprehensive femoral head coverage. Failure to adequately correct posterior acetabular deficiency may lead to posterior hip instability and increase the risk of early hip osteoarthritis [34–36].

While reported normal AV values range from 15° to 20°, dysplastic hips have typically exhibit excessive anteversion ranging from 21° to 23° [19, 23, 33]. However, in our study, the AOHD group had significantly higher acetabular anteversion (mean AV 22.2°) than the residual HD group (mean 17.3°), which did not differ significantly from the control group (mean AV 18.9°). Concurrent anterior and posterior wall deficiency may contribute to decreased AV in residual HD but it did not result in retroverted acetabulum in our series (Fig. 6). Both excessive acetabular anteversion and retroversion have been described as negative predictive factors for PAO survival rates [34].

Common deformities in the dysplastic femur include an aspheric femoral head, a valgus neck, and reduced femoral head-neck offset with increased anteversion, depending on the etiology of HD [35, 36]. In our study, the mean femoral NSA values were slightly higher in both the residual HD group and the AOHD group compared to the control group, but the differences were not statistically significant. The caput valgus in the residual HD group could be the result of lateral growth disturbance of the proximal femur in Kalamchi type II ON [25]. Among 32 residual HD cases, 17 were identified as type II ON with increased femoral NSA; however, femoral varus osteotomy was not necessary for hip deformity correction.

**Fig. 7:**
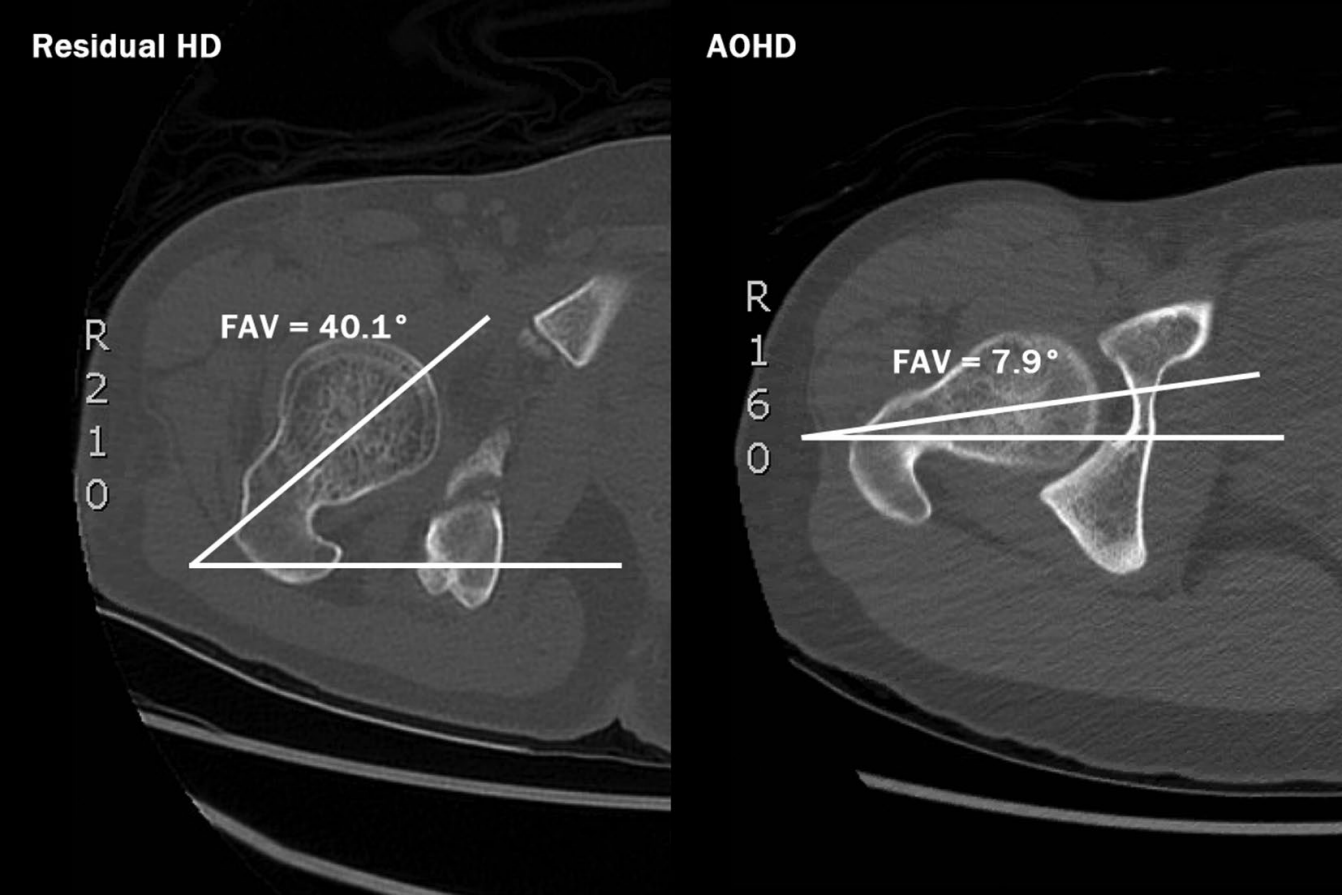
Comparison of the axial images of the sample images from the residual HD group (left) and the AOHD group (right) used to measure the FAV

For femoral version, we found significantly excessive FAV in the residual HD group compared to both the AOHD and control groups (Fig. 7; Table 5). Excessive FAV is also one of the important pathological changes of childhood DDH that may cause abnormal joint stress [37, 38]. One study found that FAV significantly correlated with acetabular version and coverage in patients with anterior and global acetabular deficiency subgroups of HD. It was also suggested that asymmetrical interaction between the femoral head and acetabulum led to dysplastic hips with anterior and global acetabular deficiency [39]. However, in our AOHD group, the mean FAV value was not significantly higher than the control group, suggesting that the two HD groups develop through distinct pathomechanisms. This aligns with findings by Sankar et al., who reported that a mean FAV of 50.3° with a standard deviation of 17.9° in 37 consecutive pediatric DDH cases [37]. When considering proximal femoral abnormalities in residual HD, it is essential to assess concurrent rotational malalignment of both the acetabulum and femur to determine the need for femoral deformity correction alongside the acetabular procedure for optimal hip biomechanics.

In this study, we hypothesize that different underlying pathomechanisms contribute to residual HD and AOHD. A genetic etiology has been proposed for developmental dysplasia of the hip, with several genetic variations in the VDR and ESR1 genes being associated with an increased risk of DDH and abnormal acetabular morphology [1–3]. Future studies investigating genetic differences between these two patient groups may provide further insight into their distinct pathomechanisms.

### Limitations

First, the retrospective nature of this study introduces a potential risk of selection bias. To mitigate this, we included consecutive HD patients who underwent PAO during the study period, along with a normal control group, ensuring that our findings are representative of both groups underwent PAO.

Although the relatively small case numbers, particularly in the residual HD group, may reduce the statistical reliability and generalizability of our result, however, this distribution accurately reflects our cohort. Thus, we believed these findings are representative of both HD groups treated with PAO.

Second, previous studies have demonstrated a positive correlation between CT-measured femoral torsion (AV) and radiographic parameters such as AHI and HLI. Additionally, low-dose biplanar radiographs (BPR) have been shown to reliably calculate 2D and 3D acetabular coverage in comparison to CT imaging. Given these findings, the necessity of routine CT imaging should be re-evaluated to minimize radiation exposure [40, 41].

Third, due to the indication for CT and ethical concerns regarding radiation exposure, FAV measurement was not referred to of the femoral condyle plane. However, since the knee was positioned straightforwardly during the CT scan, any inaccuracies should be minimal.

Finally, although our data revealed various radiographic morphological features in residual HD and AOHD, their clinical consequences and impact on surgical reconstruction remain to be fully determined.

## Conclusions

Our analysis of 3D hip morphology in a consecutive series demonstrated that both residual and AOHD patients exhibits typical characteristics of multiplanar acetabular deficiency. However, residual HD is characterized by lower acetabular volume, greater femoral head lateralization, poorer posterior acetabular support and excessive FAV compared to AOHD cases. These findings provide valuable guidance for the surgical correction of the two distinct HD groups. While standard PAO surgery is suitable for addressing AOHD patients, managing residual HD should focus more on enhancing global coverage by increasing lateral and internal rotation, as well as medialization of the joint center. Caution is necessary with anterior tilt adjustment to avoid excessive anterior acetabular coverage and iatrogenic retroversion. Excessive FAV can also compromise hip joint stability and function. The relation of femoral anteversion and the posterior acetabular deficiency must be considered and managed correctly. Finally, the long-term outcome of DDH treatment in childhood remained uncertain until skeletal maturity is reached.

## Data Availability

No datasets were generated or analysed during the current study.

## Abbreviations

HD: Hip dysplasia
DDH: Developmental dysplasia
AOHD: Adolescent-onset hip dysplasia
ON: Osteonecrosis
PAO: Bernese periacetabular osteotomy
LCEA: Lateral center-edge angles
ACEA: Anterior center-edge angles
TA: Tönnis angle
ADR: Acetabular depth ratio
AHI: Acetabular head index
HLI: Head lateralization index
AV: Acetabular version
AASA: Anterior acetabular sector angles
PASA: Posterior acetabular sector angles
NSA: Femoral neck shaft angle
FAV: Femoral neck anteversion
ICC: Intraclass correlation coefficient
ANOVA: Analysis of variance
